# Publication of clinical trials on medicinal products: follow-up on trials authorized in Hungary

**DOI:** 10.1186/s13063-022-06268-y

**Published:** 2022-04-21

**Authors:** Kinga Amália Sándor-Bajusz, Andrea Kraut, Odgerel Baasan, Gergely Márovics, Károly Berényi, Szimonetta Lohner

**Affiliations:** 1 Cochrane Hungary, Clinical Centre of the University of Pécs, Medical School, University of Pécs, Pécs, Hungary; 2 Doctoral School of Clinical Neurosciences, Medical School, University of Pécs, Pécs, Hungary; 3 Doctoral School of Law, Faculty of Law, University of Pécs, Pécs, Hungary; 4 Doctoral School of Health Sciences, University of Pécs, Pécs, Hungary; 5 Department of Public Health Medicine, Medical School, University of Pécs, Pécs, Hungary

**Keywords:** Trial registration, Publication rates, Time to publication, Authorship, Research impact, Evidence-based medicine

## Abstract

**Background:**

Clinical research should provide reliable evidence to clinicians, health policy makers, and researchers. The reliability of evidence will be assured once study planning, conducting, and reporting of results are transparent. The present research investigates publication rates, time until publication, and characteristics of clinical trials on medicinal products associated with timely publication of results, measures of scientific impact, authorship, and open access publication.

**Methods:**

Clinical trials authorized in Hungary in 2012 were followed until publication and/or June 2020. Corresponding scientific publications were searched via clinical trial registries, PubMed (MEDLINE), and Google.

**Results:**

Overall, 330 clinical trials were authorized in 2012 of which 232 trials were completed for more than 1 year in June 2020. The proportion of industry initiation was high (97%).

Time to publication was 21 (22) months [median (IQR)]. Time to publication was significantly shorter when trials involved both European and non-European countries (26 vs 69 months [median]; hazard ratio = 0.38, 95% CI 0.22–0.66, *p*< 0.001), and were registered in both EU CTR and clinicaltrials.gov (27 vs 88 months; hazard ratio = 0.24, 95% CI 0.11–0.54; *p*< 0.001) based on survival analyses.

A significant amount (24.1%) of unpublished clinical trial results were accessible in a trial register. The majority of available publications were published “open access” (70.93%). A minority of identified publications had a Hungarian author (21.5%).

**Conclusions:**

We encourage academic researchers to plan, register and conduct trials on medicinal products. Registries should be considered as an important source of information of clinical trial results. Publications with domestic co-authors contribute to the research output of a country. Measurable domestic scientific impact of trials on medicinal products needs further improvement.

**Supplementary Information:**

The online version contains supplementary material available at 10.1186/s13063-022-06268-y.

## Introduction

Clinical research should provide reliable evidence to clinicians, health policy makers, and researchers [1]. This is achieved once results are made publicly available [2]. On a national level, published research means that the resources expended are not waisted and results become part of the international medical knowledge. Published research with a domestic co-author contributes to the assessment of the scientific performance of a country.

As of 2014, registration in the European Union Clinical Trials Register (EU CTR) administered by the European Medicines Agency (EMA) is mandatory for all trial of any medicinal product conducted within a member state of the European Union (EU). The 2012 European Commission guideline 2012/c302/03 requires sponsors to ensure all trials registered on EU CTR disclose their results to the EMA within 12 months of trial completion; phase I trials are exempt unless they are part of a pediatric investigation plan [3]. Voluntary initiatives [4] and recommendations [5] have begun to emphasize the importance of registration of clinical trials and subsequent reporting of results. The EU CTR also attempts to increase awareness on mandatory posting of results [6] and has recently launched a page with a tutorial to facilitate the posting of results on the EU CTR webpage [7].

Beyond mandatory posting in the EU CTR, many European researchers additionally register their studies on ClinicalTrials.gov, the largest trial register worldwide. It remains unclear if these “multiple registrations” are an intent to improve the transparency and whether they are associated with a more likely publication of trial results.

Results posted in registries have limited impact and awareness in the scientific community. Study results in registries appear in a standardized format which facilitates data provision [8, 9]; however, these results do not undergo rigorous evaluation as do full scientific publications during the peer review process. Besides, publications and scientometrics are currently—despite international initiatives to change this [10]—an integral part of research evaluation and play a crucial role in decision making for national research policies, funding, promotions, and the careers of scientists [7]. Results posted in registries do not contribute to the total research output of either the participating researchers or the participating country.

The aim of this methodological cohort study was to investigate the visibility of authorized medical research conducted in a given country. The study equally analyzed how this visibility affects the research output of that country. We investigated publication rates, time until publication, and the relationship between results posted in trial registers and published as full scientific publications. Further, we aimed to identify trial characteristics associated with timely publication of trial results, measures of scientific impact, authorship, and open access publication in a representative sample of clinical trials authorized in Hungary.

## Methods

### Search strategy

We used the advanced search function of the EU Clinical Trials Register (www.clinicaltrialsregister.eu) to identify clinical trials registered from January 1, 2012, to December 31, 2012, with Hungary as a participating research center.

### Inclusion and exclusion criteria

Clinical trials were eligible for our study if (a) Hungary was a site of the clinical trial, (b) trials were registered in EU Clinical Trials Register by the responsible Hungarian authority (National Institute of Pharmacy, Hungary) in 2012, and (c) no restrictions were applied to the trial phase, trial status or participant characteristics (e.g., age, gender, disease group).

### Identification of trials in clinicaltrials.gov and data extraction from registries

We extracted the following pre-defined study characteristics from EU CTR: full title of the trial, authorization date, trial start and completion, information on participating countries, sponsor, funder, trial scope, trial design, blinding, sample size, study phase, therapeutic area and presence of a data monitoring committee (DMC).

We tried to identify included trials in the register clinicaltrials.gov by searching the EU CTR identifier or by the use of specific PICO terms.

We determined whether study results were available in the study registries EU Clinical Trial Register and ClinicalTrials.gov. In this current paper, we aim to distinguish results available in the registries (“results in registries”) from results published as full scientific publications (“publication”).

### Identification of corresponding scientific publications

Full scientific publications were defined as papers published in any scientific journal and reporting study results on pre-defined outcomes. We excluded methods papers, published protocols and publications which reported results of a secondary analysis.

The first screening was performed in February 2019 and then 16 months later in June 2020. Publications were identified in a step-by-step process for each trial. First, we checked whether publications were already added to the register. In a second step, we searched for publications in the PubMed database with the following identification data: (a) the trial register number, (b) the investigators’ names, and (c) keywords describing the intervention or the condition (PICO elements). The third step involved a Google search with the same search terms.

All identified publications belonging to the registered study were checked for their content (study design, population characteristics, dates of recruitment, intervention, comparator). Publications which clearly described the results of the originally planned and registered study were included.

### Data extraction from scientific publications

We extracted the following data from the identified publications: the presence of author(s) with a Hungarian affiliation; the number of Hungarian authors or whether Hungarian participation in the study was mentioned in a way other than author affiliation; the journal’s name, and date of publication. In cases when there were different forms of publishing (e.g., published electronically ahead of print), we recorded the first date when the full text of the final manuscript was accessible.

To estimate the time to publication, we counted the total months elapsed between trial end date available in the EU Clinical Trials Register and the publication date. We expressed publication rate as the percentage of clinical trials with a full scientific publication divided by all clinical trials. The number of all clinical trials was calculated separately for each month after trial completion.

Impact factors for each journal were derived from the Journal Citation Report, Clarivate Analytics via www.webofknowledge.com. Scimago journal rank (Q1–Q4) was derived from www.scimagojr.com.

We also wanted to determine the public’s access to the results of published scientific papers. We therefore investigated whether scientific publications were published openly or with closed access. No distinctions were made between publications published in an open access journal and publications published in a hybrid journal by using the open access option.

### Statistical analysis

Results were summarized as frequencies and proportions for binary data, and as medians and interquartile ranges for continuous data. We considered three analysis sets: a dataset based on all trials authorized in 2012 in Hungary, a dataset based on the trials completed for longer than one year in June 2020, and a dataset based on corresponding publications. Data was analyzed by descriptive statistics and cross-tabulation. Time to publication was estimated with the nonparametric Kaplan-Meier estimator and the logrank test (by Mantel-Cox) was used to estimate the potential effects of investigated factors on time to publication. Hazard ratios and confidence intervals were calculated on the basis of a Cox regression model. We used the statistical program SPSS version 26 (SPSS INC., Chicago, IL, USA) for our analyses.

## Results

### Included clinical trials

A total of 614 clinical trials were identified in our search. Trials were excluded if the year 2012 did not correspond to the Hungarian registration date, but to the registration date of another participating country of multinational trials. A total of 330 Hungarian national or international clinical trials were eligible for inclusion into our methodological cohort (see Additional file 1). Eight years after trial authorization (in June 2020), 232 were “completed” trials for at least 1 year. Baseline characteristics of these trials are presented in Table 1.

**Table 1:** Baseline characteristics of investigated studies

	All trials authorized in 2012 (*n*=330)	Trials authorized in 2012 and completed for more than one year in 2020 (*n*=232)
	%	%
**Number of involved countries (one or more)**		
- National trial - International trial	3.03 96.96	1.72 98.28
**Sponsor**		
- Industry - Non-industry	96.96 2.72	98.7 1.3
**Funder**		
- Industry - Non-industry - Not clear	94.24 1.51 4.24	94.83 0.43 4.74
**Therapeutic area**		
- Infectious diseases - Cancer - Musculoskeletal disorders - Gastrointestinal diseases - Respiratory tract diseases - Nervous system diseases - Cardiovascular diseases - Nutritional and metabolic diseases - Immune system diseases - Other	5.45 22.72 10.9 8.48 7.57 8.48 7.87 7.87 8.18 13.28	6.47 20.26 11.64 8.62 8.19 7.33 5.60 9.05 8.19 14.66
**Registration**		
- Registered in EU-CTR only	10.30	8.19
- Registered in both EU-CTR and ClinicalTrials.gov	89.70	91.81

Most of the trials were international, initiated, and funded by the industry. The majority assessed both efficacy and safety of a therapeutic intervention. Of the investigated clinical trials, 91.8% were registered not only in EU CTR, but both in the EU CTR and the clinicaltrials.gov database.

### Publication rates

**Fig. 1:**
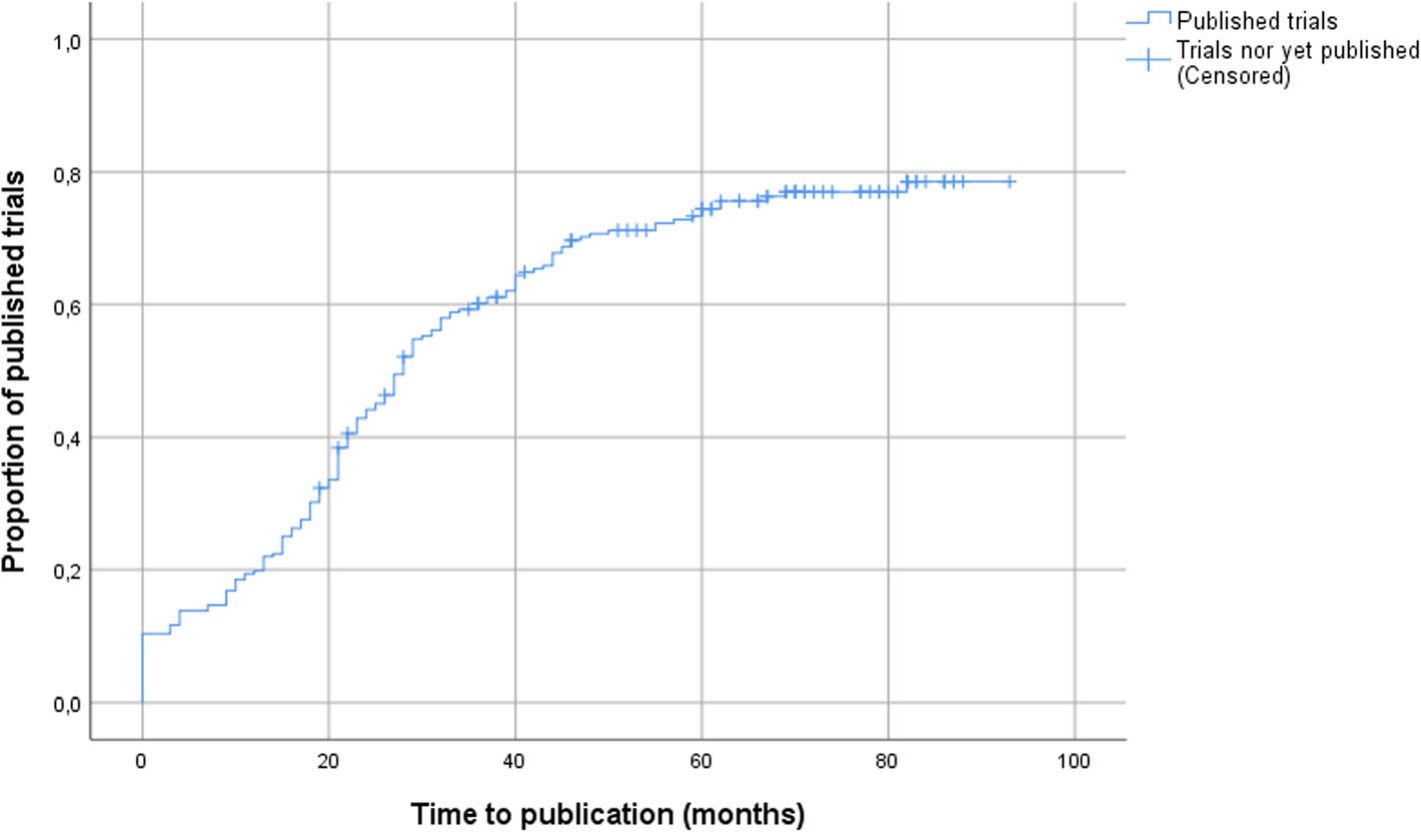
Publication rates over time in a cohort of trials authorized in 2012 in Hungary (*n*=232)

Publication rate over time is shown in Fig. 1.

Twelve months after completion, 19.8% of clinical trials were published as a full scientific paper. While five years after trial completion 19.4% of studies were still not available as a full publication. The time between the end of the clinical trial and the publication of the full scientific paper was 21 (22) months [median (IQR)].

At the time of our search, 74.1% of completed clinical trials had an available corresponding scientific publication. Overall, 70.7% of trial results were available as both full scientific publications and were posted in registries; 3.4 % were publications without posted results in registries, 24.1 % were in registries without an available full scientific publication, and 1.7 % were completed for longer than a year without available results (see Additional file 2).

### Factors influencing time to publication

Time to publication was significantly shorter for trials conducted both inside and outside of Europe compared to trials only in Europe (26 vs 69 months [median]; Additional file 3; log-rank *p*< 0.001); and in case of trials registered in both EU CTR and clinicaltrials.gov compared to trials registered in EU CTR only (27 vs 88 months [median]; Additional file 4; *p*< 0.001). Time to publication was not influenced by either being a national or an international trial (37 vs 27 [median]; Additional file 5; *p*=0.45), an RCT or non-RCT (27 vs 32 [median]; Additional file 6; *p*=0.56), or by the presence or lack of a DMC (25 vs 33 [median]; Additional file 7; *p*=0.28) (Table 2).

**Table 2:** Predictors of time to publication

	Time to publication in trials with available corresponding publication (***n*** = 172) (months [mean (SE)]	Time to publication in trials completed for longer than one year (***n*** = 232) (months [mean (SE)]	HR (95%CI)*	***p***
**Trial sites**				
Only Europe	34.86 (4.56)	64.28 (5.28)	1	
Also from outside Europe	21.85 (1.31)	35.00 (2.12)	0.38 (0.22–0.66)	0.001
**Trial registration**				
In EU CTR only	29.00 (9.53)	68.14 (6.81)	1	
EU CTR and clinicaltrials.gov	22.69 (1.29)	36.43 (2.16)	0.24 (0.11–0.54)	0.001
**Participating countries**				
National	42.33 (7.42)	55.00 (11.87)	1	
International	22.57 (1.29)	38.14 (2.08)	0.65 (0.21–2.04)	0.46
**Trial design**				
RCT	22.59 (1.43)	37.87 (2.28)	1	
Non-RCT	23.28 (2.90)	42.24 (5.27)	1.12 (0.75–1.68)	0.57
**DMC**				
No	27.93 (2.01)	42.48 (2.99)	1	
Yes	17.81 (1.40)	36.08 (3.01)	0.85 (0.63–1.15)	0.29

*Factors influencing time to publication were investigated in the dataset based on the trials completed for more than 1 year in June 2020 (*n*=232)

**Fig. 2:**
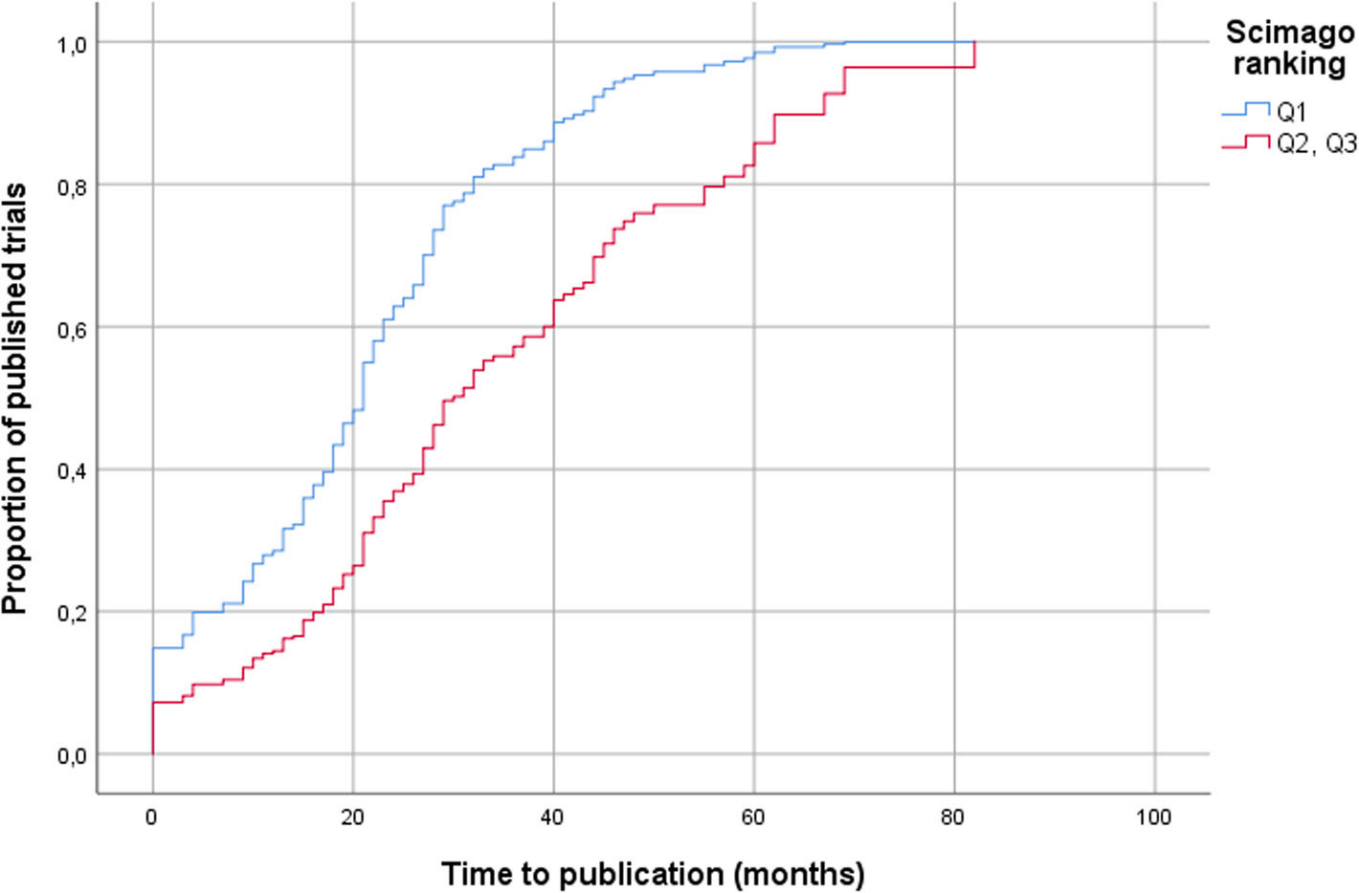
Publication rates over time in Q1 (*n*=151), and Q2–Q3 (*n*=21) journals. The Q2–Q3 group contains 19 Q2 and two Q3 publications

Scientific results were published earlier, if published in a Q1 as compared to a Q2–Q3 journal (Fig. 2; log-rank *p*=0.001; HR [95% CI]: 2.14 [1.32–3.48], *p*=0.002).

### Measures of scientific impact

#### Hungarian authorship and participation

Hungary was mentioned in 48.0% of the scientific publications (either as an author’s affiliation or as a study site listed in the text). Publications had at least one author with a Hungarian affiliation in 21.5% of cases (16.3% with one Hungarian author, 5.2% with two or more Hungarian authors).

Trials conducted within Europe (RR 2.184 [1.104–4.321]) and trials initiated by the academy instead of the industry (RR 3.706 [1.953–7.032]) significantly increased the probability of a scientific publication with a Hungarian author. Other investigated factors, such as studies registered in EU CTR only (0.769 [0.125–0.707), the lack of a DMC (1.071 [0.594–1.933]), non-RCT studies (1.216 [0.588–2.511]), and national trial studies (1.565 [0.308–7.957), had no effect on Hungarian authorship.

#### Impact factor and Scimago ranking of the journal

No differences were observed between the impact factor of publications with a Hungarian author compared to publications without (IF16.02 [16.59] vs. 19.47 [23.89]; mean [SD]; *p*=0.389). The impact factor tended to be higher for international compared to national trials (18.98 [22.63] vs. 4.58 [4.48]; *p*=0.171), for trials conducted in and outside of Europe compared to trials within Europe only (19.28 [22.77] vs.12.47 [19.09]; *p*=0.264), and for trials registered in both the clinicaltrials.gov and the EU CTR compared to trials registered in EU CTR only (19.08 [22.80] vs.8.92 [8.21]; *p*=0.445).

Trials with a data monitoring committee (DMC; i.e., a group of experts external to the study monitoring safety during study conduct) were more likely to publish results in a Q1 journal (1.131 [1.008–1.270]), while studies initiated by the industry were less likely (0.875 [0.826–0.926]). Other investigated factors, such as registration in two or only one registry (1.055 [0.735–1.516]), international versus national trial (2.663 [0.537–13.204]), trials conducted outside of versus within Europe (1.249 [0.893–1.748]), or study design (1.020 [0.871–1.195]) did not affect the Scimago journal ranking of the subsequent publication.

#### Study results available for all, as part of an open access publication

A total of 70.93% of scientific publications were open access, making the results accessible to the public. None of the investigated factors increased the probability for open access publication (see Additional file 8).

### Influence of the industry on transparency and scientific impact of a trial

The number of authorized clinical trials initiated by the academy was extremely low (2.7% of all authorized trials). Clinical trials initiated by the industry or the academy are shown and compared in Table 3.

**Table 3:** Role of industry in trial conduct: comparison between industry-initiated and academy-initiated clinical trials (*n*=329; no information for 1 trial)

	Industry-initiated clinical trials (***n*** = 320) %	Investigator-initiated clinical trials (***n*** = 9) %
**Financial background**		
Founded by the industry	96.88	11.11
Founded by non-industry	0	55.55
No information available	3.13	33.33
**Trial design**		
RCT	79.69	88.88
Non-RCT	16.88	11.11
No information available	2.81	0
**Blinding**		
Double-blind	62.19	44.44
Single-blind	1.25	0
Open-label	21.88	44.44
Not clear	14.69	11.11
**Collaboration**		
National	1.88	44.44
International (only EEA)	88.75	33.33
International (within and outside the EEA)	9.375	22.22
**Trial scope**		
Safety and efficacy	93.75	55.55
Safety	5	0
Efficacy	1.25	33.33
None	0	11.11
**Trial phase**		
Phase I	1.5625	0
Phase II	38.75	0
Phase III	52.8125	55.55
Phase IV	4.0625	22.22
Not mentioned/More phases	2.8125	22.22
**Data monitoring committee**		
Yes	55.31	33.33
No	44.69	66.66
**Availability of study results**		
Results posted in EU CTR	73.125	11.11
Results published as full scientific publication	62.8125	55.55
Time to publication (months; mean [SD])	21.70 [16,82]	18.33 [3,77]
**Impact on the scientific reputation of the authorizing country**		
Publication with at least one Hungarian author	12.1875	33.33
Publication mentioning Hungarian participant(s)	27.5	44.44

Industry-initiated trials with accessible information regarding funding were all funded by the industry. Investigator-initiated clinical trials were also partly funded by the industry. Information on funding was unavailable for one third of investigator-initiated trials.

A DMC was available to a much larger extent in trials initiated by the industry.

Results of investigator-initiated trials were significantly less likely to be posted in a clinical trial register (73.1% vs. 11.1%) and also slightly less likely to be published as a full scientific publication (62.8% vs. 55.6%).

Both the rate of publications with at least one Hungarian author (33.3% vs. 12.2%), and the rate of publications mentioning Hungarian participation in any form (44.4% vs. 27.5) was higher among investigator-initiated as compared to industry-initiated trials.

## Discussion

### Summary of findings

Our study provides empirical evidence about the publication tendencies of authorized clinical trials in Hungary and their impact on the scientific reputation of the authorizing country.

Overall, 97.3% of authorized and EU CTR-registered clinical trials were initiated by the industry. About 20% of clinical trials were published within one year after trial completion. Trials conducted only within Europe and registered only in the EU CTR register were published significantly later.

Universality is a fundamental principle of science [11]; open access publications have therefore the largest impact on the scientific community. In this study, 70.93% of publications were found to be openly accessible to the public. However, we were not able to identify trial characteristics which might influence the access to scientific publications.

Only 21.5% of publications included Hungarian co-authors. Hungarian authors were most often present in publications of clinical trials conducted in Europe and trials initiated by the academy.

Trials registered both in the EU Clinical Trials Register and clinicaltrials.gov database were more likely to result in full publications. Almost one quarter of the results of the investigated clinical trials were available in registries, but not as a full scientific publication. Only a minority of clinical trials were available as full scientific papers and without results posted in registries.

### Strength and limitations of this methodological research study

Our study analyzed a representative sample of trials authorized in Hungary which allows the results to be generalized to the country. All trials were registered in study registries, thus basic study information was available for all the trials included in our study. All data extractors were trained and the main outcome data were double-checked and double-extracted.

Our study has limitations. The cohort was composed of trials that were authorized and included in the EU Clinical Trials Register by the national authority. Due to the low number of investigator-initiated trials, it is difficult to draw firm conclusions on their publication tendencies. Nevertheless, our results may indicate a publication trend also for investigator-initiated trials.

The EU Clinical Trials Register defined trials as completed when a trial „has been completed in accordance with the full requirements of the protocol”, which might be interpreted differently by researchers and may have impacted our results. We searched for scientific publications in 2020 and trials completed within one year before the search date were excluded.

### Comparison with other studies

To the best of our knowledge, this is the first study that investigates to what extent research authorized and conducted in a given country becomes visible and affects t domestic scientific performance.

Authorship issues were previously discussed by several papers, analyzing gender distribution of authorship [12, 13]; association between sponsorship and authorship [14, 15]; under-representation of researchers from specific regions in papers published from research done in these regions [16, 17]; and difficulties and possibilities in determining authorship in multicenter clinical trials [18, 19]. These studies were mostly based on publication data sets. We demonstrate a new approach to prospectively follow trials authorized in a given country until publication and investigate authorship in this matter.

We found one large cohort study that investigated compliance with result reporting in the EU Clinical Trials Register up to December 2016 [8]. Trials with a commercial sponsor more likely posted results in the EU CTR than studies with a non-commercial sponsor (68.1% v 11.0%) [8]. This is in line with the results of the present study: industry-initiated trials posted significantly more results in the EU CTR (73.1%) than investigator-initiated trials (11.1%). Investigator-initiated trials are in need of standardized procedures [20], and the proper implementation of the following additional requirements: periodic quality control assessments during trial implementation [21], improved reporting about funding [22], improvement of reporting [23], mandatory trial registration and trial result posting.

### Implications of findings for practice, policy and future research

All interventional clinical trials on medicinal products authorized in the European Union—without any distinction by type of sponsor—should be registered in the EU Clinical Trials Register as a first step. Trial registration in clinicaltrials.gov can further increase the visibility of registered European trials.

The present research revealed that a surprisingly low number of clinical trials initiated by the academy get registered in the EU Clinical Trial Register in Hungary. Academic clinical trials have an important place on the map of clinical research. These studies focus on specific questions that arise during clinical care and are extremely important in everyday medical practice. These include but are not limited to facilitating the optimization of a therapy, or the discovery of potential new clinical areas where a therapeutic intervention can be used. Increased transparency of results of academic clinical trials is essential for evidence-based medical decision-making and optimal patient management.

Posting trial results in study registries might be the first step to allow study results to become openly available for the public; however, results should be additionally published as soon as possible as a scientific publication after trial completion. Systematic reviewers and guideline developers are advised to search clinical trial registers in addition to electronic databases to identify study results, which have not been published as full text publications at the time of the search.

The participation of Hungarian researchers in industry-initiated studies on medicinal products has only partial measurable scientific benefits, since Hungarian researchers appear as authors in only a fraction of scientific publication derived from these trials. Several publications did not even include the list of countries of trial participants. The Lancet journals strongly support the inclusion of domestic authors in papers reporting studies from those countries; we also would like to “encourage authors to include researchers who originally collected the data, where possible, and to share expertise in analysis and other skills, so that the research capacity of the country from which the data were obtained is strengthened” [24], i.e., to enable local researchers to fulfill the criteria for authorship developed by the International Committee for Medical Journal Editors.

The scientific performance of universities and countries is evaluated and ranked—despite valuable initiatives for change—based on research productivity (i.e., the number of scientific publications), research impact, and research excellence (i.e., the number of scientific papers in high-impact journals). Slightly over a fifth of authorized Hungarian trials result in scientific publications with a Hungarian co-authorship. We can conclude that the authorized, mainly industry-initiated clinical trials on medicinal products currently result in limited measurable scientific benefits to the participating researchers and their countries.

## Conclusions

We call researchers of investigator-initiated clinical trials to register their trials in an openly available clinical trial register. Trial registers have to be considered as an important source of information of clinical trial results, as they may contain results from unpublished trials or trials published with closed access. Domestic scientific impact of trials on medicinal products has to be further improved. An increase in the number and role of investigator-initiated trials might help to achieve this goal.

## Supplementary Information

**Additional file 1.** Flowchart of clinical trial selection

**Additional file 2.** Information transfer process from trial authorization until the publication of trial results

**Additional file 3.** Publication rates over time in clinical trials involving only European and both European and non-European countries

**Additional file 4.** Publication rates over time in clinical trials registered in both EU CTR and clinicaltrials.gov

**Additional file 5.** Publication rates over time in national and international clinical trials

**Additional file 6.** Publication rates over time according to trial design

**Additional file 7.** Publication rates over time in clinical trials with and without a data Monitoring Committee

**Additional file 8.** Probability of open access publication

## Data Availability

The data supporting the conclusions of this article is included within the article (and its additional files).

## References

[1] Ioannidis JP (2016). Why most clinical research is not useful. PLoS Med.

[2] DeVito NJ, Goldacre B (2019). Catalogue of bias: publication bias. BMJ Evidence Based Med.

[3] Commission Guideline — Guidance on posting and publication of result‐related information on clinical trials in relation to the implementation of Article 57(2) of Regulation (EC) No 726/2004 and Article 41(2) of Regulation (EC) No 1901/2006 (2012/C 302/03). Available at: https://eur-lex.europa.eu/legal-content/EN/TXT/PDF/?uri=CELEX:52012XC1006(01)&from=EN. Accessed 08 Apr 2022.

[4] Brown T (2013). It’s time for alltrials registered and reported. Cochrane Database Syst Rev.

[5] Taichman DB, Sahni P, Pinborg A, Peiperl L, Laine C, James A (2017). Data sharing statements for clinical trials: a requirement of the International Committee of Medical Journal Editors. PLoS Med.

[6] Joint Letter by the European Commission, EMA and HMA (2019). Letter to stakeholders regarding the requirements to provideresults for authorised clinical trials in EudraCT.

[7] Bornmann L, Leydesdorff L (2014). Scientometrics in a changing research landscape: bibliometrics has become an integral part of research quality evaluation and has been changing the practice of research. EMBO Rep.

[8] Goldacre B, DeVito NJ, Heneghan C, Irving F, Bacon S, Fleminger J (2018). Compliance with requirement to report results on the EU Clinical Trials Register: cohort study and web resource. BMJ (Clinical research ed).

[9] Turner L, Shamseer L, Altman DG, Schulz KF, Moher D (2012). Does use of the CONSORT Statement impact the completeness of reporting of randomised controlled trials published in medical journals? A Cochrane review. Syst Rev.

[10] San Francisco Declaration on Research Assessment. Available at: https://sfdora.org/read/ (accessed on 01 Febr 2021).

[11] Schiltz M (2018). Science Without Publication Paywalls: cOAlition S for the Realisation of Full and Immediate Open Access. PLoS Med.

[12] Xu GM, Zavalkoff S, de Wildt SN, Duffett M (2020). Gender and Authorship in Pediatric Critical Care Randomized Control Trials. Pediatric critical care medicine : a journal of the Society of Critical Care Medicine and the World Federation of Pediatric Intensive and Critical Care Societies.

[13] Bernardi K, Lyons NB, Huang L, Holihan JL, Olavarria OA, Martin AC (2020). Gender Disparity in Authorship of Peer-Reviewed Medical Publications. Am J Med Sci.

[14] Raman S, Moraes FY, Mendez LC, Taunk NK, Suh JH, Souhami L (2018). The relationship of study and authorship characteristics on trial sponsorship and self-reported conflicts of interest among neuro-oncology clinical trials. J Neuro-Oncol.

[15] Tauber M, Paul C (2017). Authorship selection in industry-sponsored publications of dermatology clinical trials. Br J Dermatol.

[16] Mbaye R, Gebeyehu R, Hossmann S, Mbarga N, Bih-Neh E, Eteki L (2019). Who is telling the story? A systematic review of authorship for infectious disease research conducted in Africa, 1980-2016. BMJ Glob Health.

[17] Kelaher M, Ng L, Knight K, Rahadi A (2016). Equity in global health research in the new millennium: trends in first-authorship for randomized controlled trials among low- and middle-income country researchers 1990-2013. Int J Epidemiol.

[18] Whellan DJ, Kraus WE, Kitzman DW, Rooney B, Keteyian SJ, Piña IL (2015). Authorship in a multicenter clinical trial: The Heart Failure-A Controlled Trial Investigating Outcomes of Exercise Training (HF-ACTION) Authorship and Publication (HAP) scoring system results. Am Heart J.

[19] Dulhunty JM, Boots RJ, Paratz JD, Lipman J (2011). Determining authorship in multicenter trials: a systematic review. Acta Anaesthesiol Scand.

[20] Mudaranthakam DP, Phadnis MA, Krebill R, Clark L, Wick JA, Thompson J (2020). Improving the efficiency of clinical trials by standardizing processes for Investigator Initiated Trials. Contemp Clin Trials Commun.

[21] Figer BH, Sapra KP, Gogtay NJ, Thatte UM (2020). A comparative study to evaluate quality of data documentation between investigator-initiated and pharmaceutical industry-sponsored studies. Perspect Clin Res.

[22] Madeira C, Santos F, Kubiak C, Demotes J, Monteiro EC (2019). Transparency and accuracy in funding investigator-initiated clinical trials: a systematic search in clinical trials databases. BMJ Open.

[23] Landewé RB, Smolen JS, Weinblatt ME, Emery P, Dougados M, Fleischmann R (2014). Can we improve the performance and reporting of investigator-initiated clinical trials? Rheumatoid arthritis as an example. Ann Rheum Dis.

[24] The Lancet P (2018). Diversity and inclusion: from priority setting to publication. Lancet Psychiatry.

