# Supplementary figures and images for: Publication of clinical trials on medicinal products: follow-up on trials authorized in Hungary
Source: Trials. 2022 Apr 21;23:330. doi: 10.1186/s13063-022-06268-y (PMC9022244; doi:10.1186/s13063-022-06268-y)

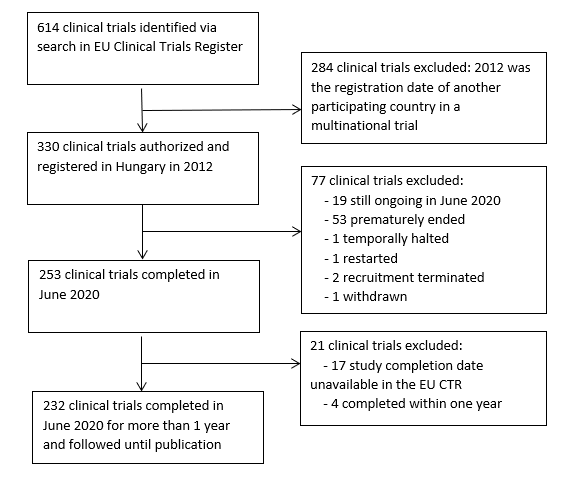

Supplement: Supplementary file 1 — Additional file 1. Flowchart of clinical trial selection [file 13063_2022_6268_MOESM1_ESM.tif]

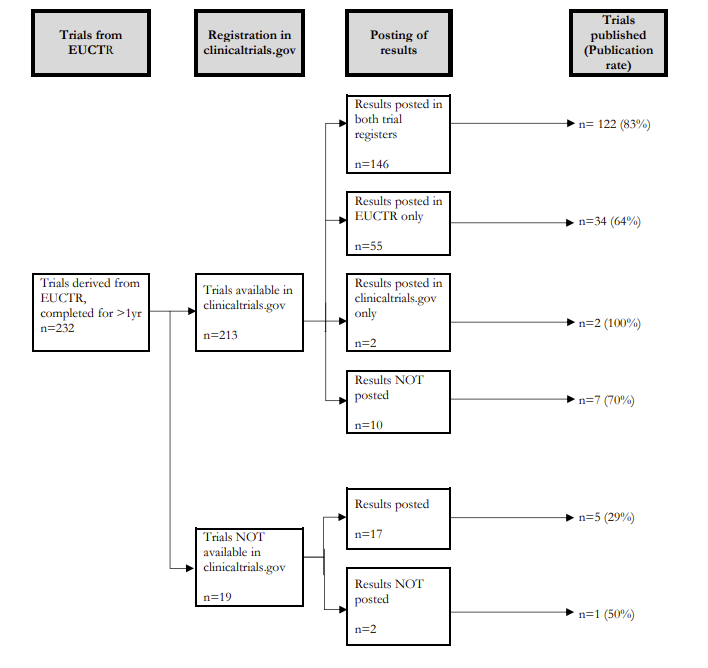

Supplement: Supplementary file 2 — Additional file 2. Information transfer process from trial authorization until the publication of trial results [file 13063_2022_6268_MOESM2_ESM.tif]

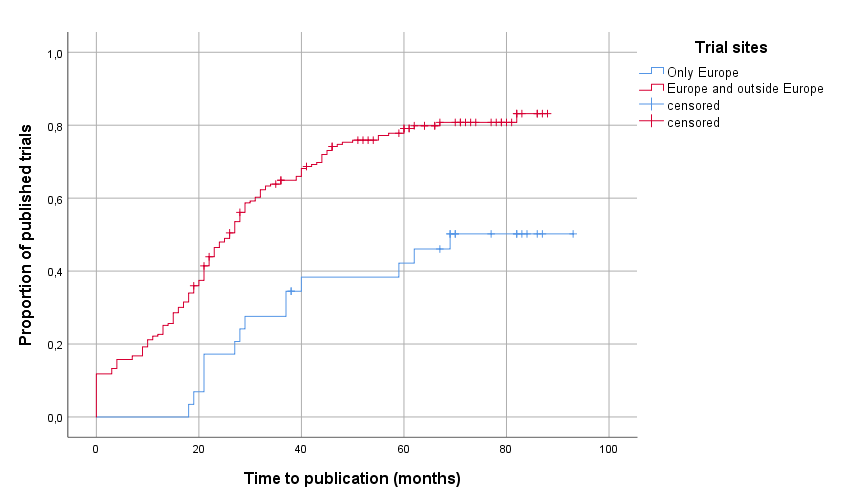

Supplement: Supplementary file 3 — Additional file 3. Publication rates over time in clinical trials involving only European and both European and non-European countries [file 13063_2022_6268_MOESM3_ESM.tif]

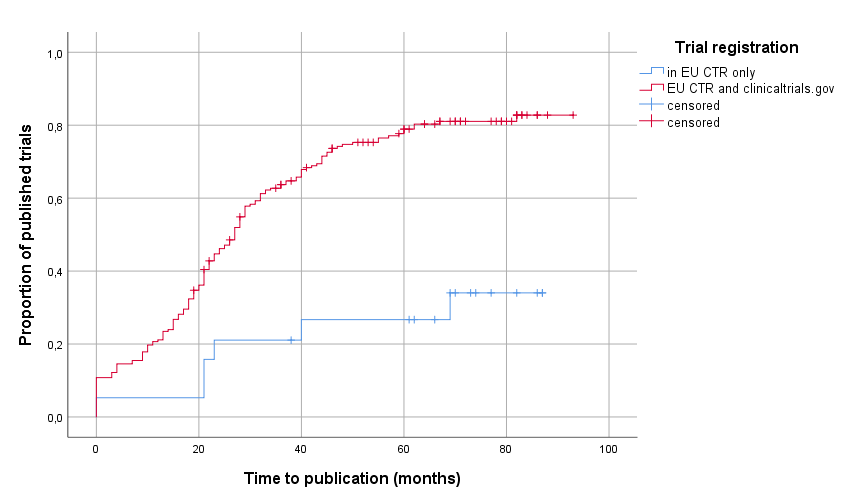

Supplement: Supplementary file 4 — Additional file 4. Publication rates over time in clinical trials registered in both EU CTR and clinicaltrials.gov [file 13063_2022_6268_MOESM4_ESM.tif]

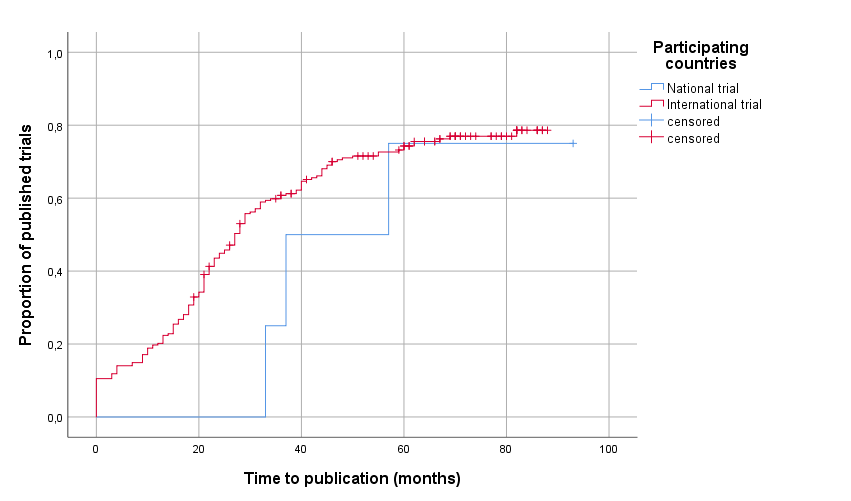

Supplement: Supplementary file 5 — Additional file 5. Publication rates over time in national and international clinical trials [file 13063_2022_6268_MOESM5_ESM.tif]

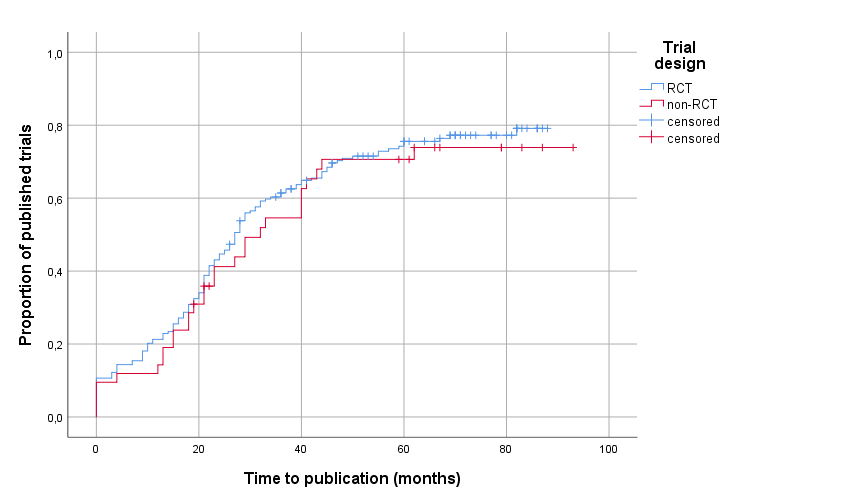

Supplement: Supplementary file 6 — Additional file 6. Publication rates over time according to trial design [file 13063_2022_6268_MOESM6_ESM.tif]

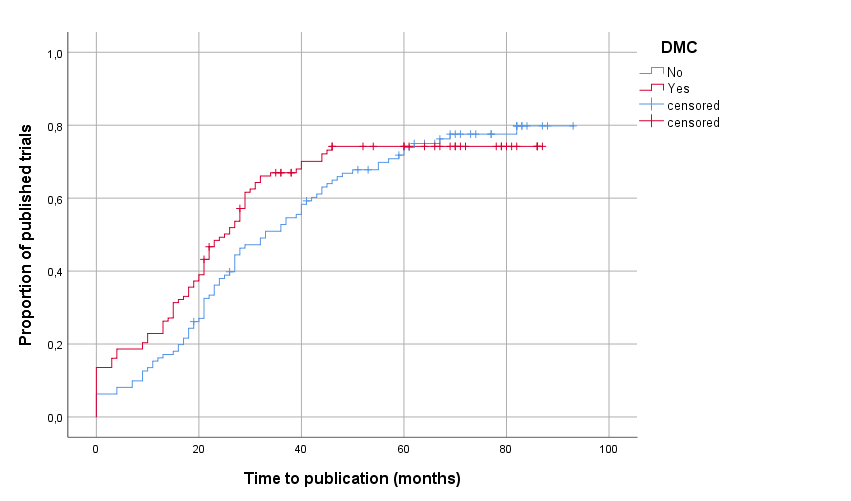

Supplement: Supplementary file 7 — Additional file 7. Publication rates over time in clinical trials with and without a data Monitoring Committee [file 13063_2022_6268_MOESM7_ESM.tif]

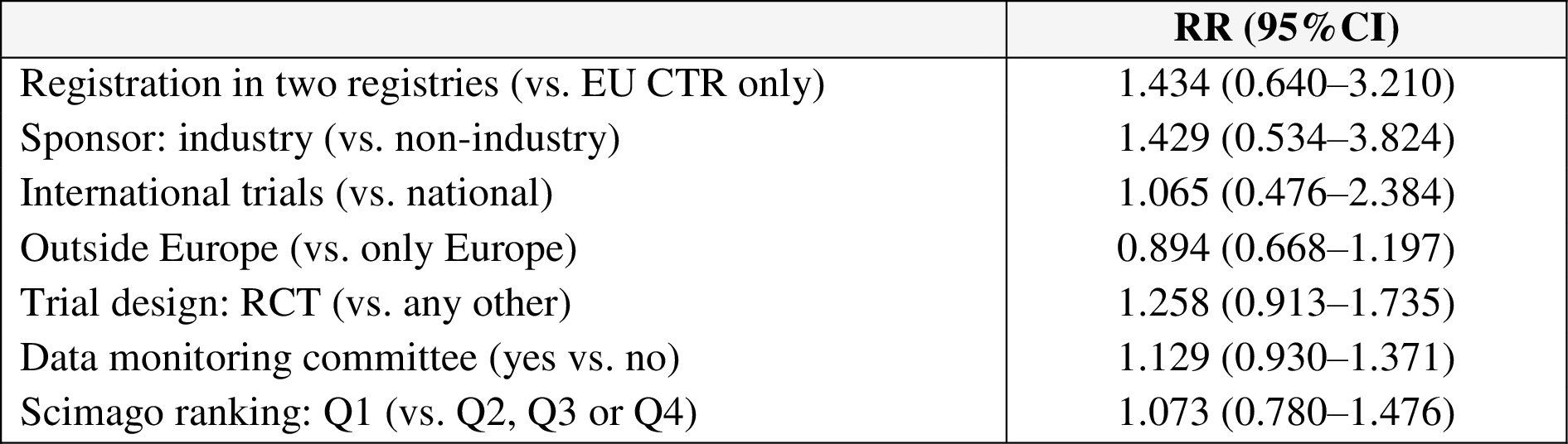

Supplement: Supplementary file 8 — Additional file 8. Probability of open access publication [file 13063_2022_6268_MOESM8_ESM.tif]
